# Two Cases Demonstrating the Role of Creeping Attachment in the Treatment of Keratinized Gingival Deficiency With Free Gingival Graft: A 12-Month Follow-Up

**DOI:** 10.7759/cureus.49537

**Published:** 2023-11-28

**Authors:** Cemre Ekşi

**Affiliations:** 1 Periodontology, Eskişehir Osmangazi University, Faculty of Dentistry, Eskişehir, TUR

**Keywords:** root coverage, keratinized gingiva, gingival recession, free gingival graft, creeping attachment, clinical attachment level

## Abstract

After free gingival graft procedure, partial or complete coverage of exposed root surfaces due to coronal migration of the gingival margin is called “creeping attachment.” This case report aimed to evaluate the results of the free gingival graft procedure performed on two patients with keratinized gingival deficiency in the mandibular anterior region and to present the creeping attachment formation process with a 12-month follow-up in light of current literature. Free gingival graft treatment was applied after the initial periodontal treatments were completed for two patients who visited the periodontology clinic complaining of gingival recession. Although the postoperative recovery was completed without any problems, it was observed that there were still root exposures in the relevant areas. Patient motivation was provided by giving oral hygiene training to the patients. After the 12-month follow-up, it was reported that denuded root surfaces were completely covered with creeping attachment formation. Complete coverage of denuded root surfaces is possible through the formation of creeping attachment, with the help of oral care and follow-up procedures, without requiring repeated surgical procedures. So, after relevant procedures, dentists must provide patients with sufficient information and awareness on this issue.

## Introduction

In a healthy periodontium, the gingival margin is clinically located at the level of the cemento-enamel junction or 1-2 mm coronal to the cemento-enamel junction [1,2]. Gingival recession is the exposure of the root surfaces due to apical migration of the gingival margin [1,2]. Gingival recession may occur due to biofilm-induced periodontal diseases, faulty tooth brushing habits, the effect of orthodontic forces, the aging process, anatomical factors such as dehiscence and fenestration, high frenulum attachment, malposition of teeth, parafunctional habits (e.g., thumb sucking, pencil biting, etc.), and external factors such as piercing [1,3]. Pedicle grafts, free grafts, or regenerative techniques are used in the treatment of gingival recession [4].

Free gingival graft treatment is a widely applied procedure to increase the width of the keratinized gingiva, cover the exposed root surface, increase the vestibule depth, remove the high frenulum attachment, and create a tissue contour where the patient can easily maintain oral hygiene [5]. Although the primary goal of free gingival graft treatment is not to cover exposed root surfaces, partial or complete coverage may occur in cases of gingival recession [6]. The average root coverage percentage with the free gingival graft procedure varies from 11% to 100% [1]. In the postoperative period, the gingival margin may advance in a coronal direction, partially or completely covering the initially exposed root surfaces. This formation, which was first defined as “creeping attachment” by Goldman, is encountered especially in the mandibular anterior region after mucogingival surgical procedures [7-9]. This newly formed tissue adheres tightly to the root surface and shows minimal sulcus depth during probing [9]. Creeping attachment is a biological phenomenon that helps prevent gingival recession and protect root surfaces. This process plays an important role in the treatment of patients who have aesthetic and functional problems with exposed root surfaces due to gingival recession [8]. In short, it has been previously reported that successful root surface coverage can be achieved by the revascularization of the graft, its adaptation to neighboring tissues, and the formation of creeping attachment [9]. It is clinically known that migration can occur slowly over a long period of time. It has been reported that this formation can occur 1 to 12 months postoperatively and can provide an average root surface coverage of 0.89-1 mm [7,9].

The current case report deals with the formation of creeping attachment, which is an effective phenomenon in improving the oral health and aesthetics of patients after a surgical procedure [8]. It aims to present the treatment, follow-up, and creeping attachment formation processes of two cases in which surgical intervention was performed to cover exposed root surfaces resulting from gingival recession in light of current literature.

This case report (only the first case) was presented as a poster at the 52nd International Scientific Congress and 30th Scientific Symposium of the Turkish Society of Periodontology on September 21-23, 2023.

## Case presentation

Case 1

**Figure 1:**
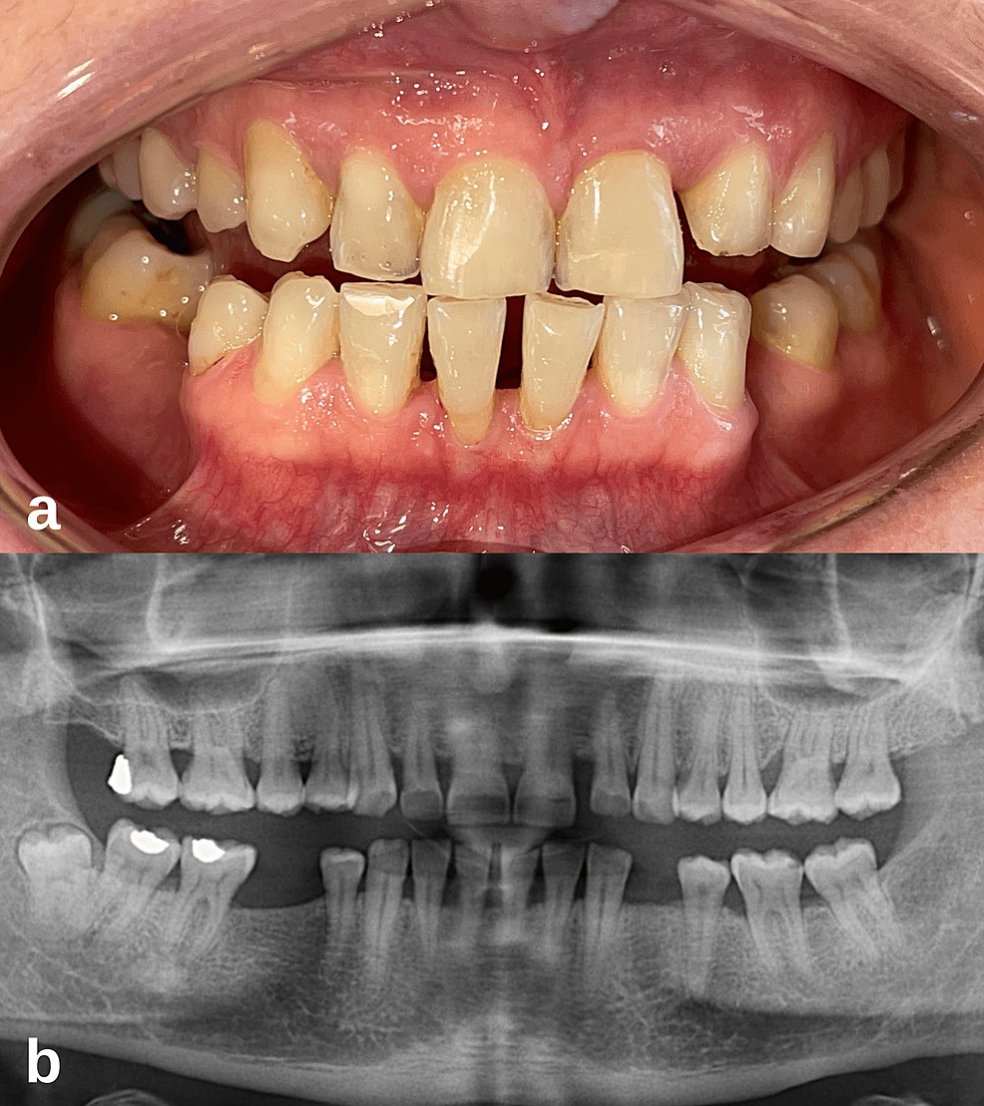
Case 1 initial periodontal examination. (a) Initial clinical intraoral image. (b) Visualization of interdental bone resorption in radiographic examination.

The clinical picture of a systemically healthy 41-year-old female patient who came to the Periodontology Clinic of Eskişehir Osmangazi University Faculty of Dentistry was consistent with gingivitis, and there was calculus on the teeth. In the clinical periodontal examination of the patient, it was determined that the keratinized gingival width of mandibular anterior teeth was inadequate (Figure 1). The gingival recession was determined by measuring millimetrically from the gingival margin to the cemento-enamel junction. The gingival recession of 1 and 3 mm was measured apicocoronally on teeth 31 and 41, respectively, with Williams periodontal probe (Nordent, CA), and the recession defects were determined as Miller’s class III [10]. In addition, 3 and 5 mm attachment losses were measured in the relevant area, respectively, and interdental bone resorption was observed in the radiographic examination (Figure 1).

In her anamnesis, the patient stated that she had sensitivity problems due to receding gingiva and that she felt aesthetically uncomfortable. It was decided to perform the free gingival graft procedure in the area to increase the width of the attached gingiva. The patient accepted the treatment, and the treatment was initiated with plaque control instructions, followed by scaling and polishing. Before the procedure, the patient’s phase I periodontal treatment was completed, and care was taken to ensure that the intraoral plaque and gingival index scores were close to zero.

**Figure 2:**
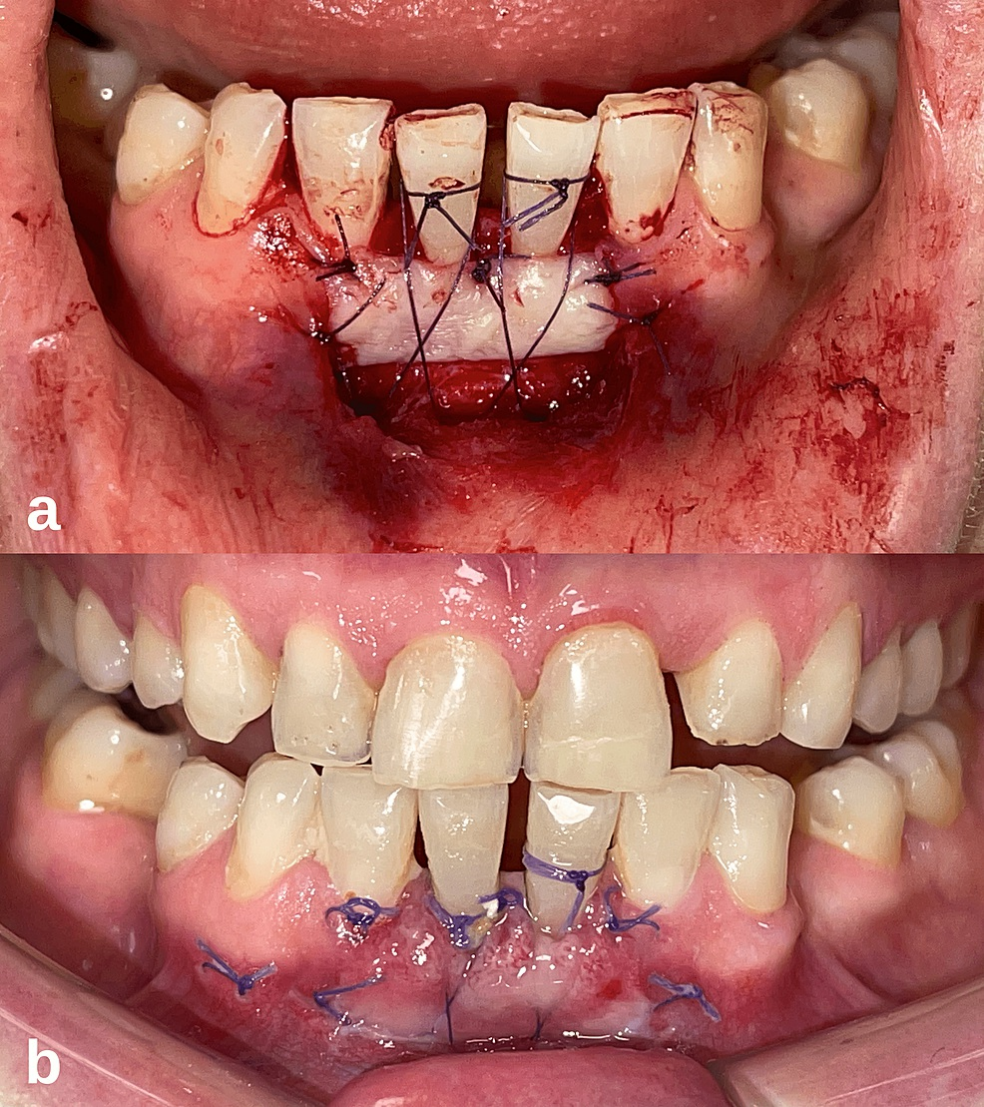
Case 1- Intraoral images after the free gingival graft procedure. (a) Fixation of the free gingival graft to the recipient site with simple and sling sutures. (b) Clinical appearance of the surgical site before the sutures were removed on the 12th postoperative day.

Local anesthesia of the anterior incisors during the surgical appointment (Maxicaine Fort, articaine hydrochloride + epinephrine bitartrate, Vem Pharmaceuticals, TR) was achieved, and root planing was performed on teeth numbered 31-41 with a Gracey 1/2 curette. A partial-thickness flap was elevated in the mandibular central incisor area with a #15 scalpel blade (Beybi, TR), and the recipient site was prepared. While preparing the recipient site, care was taken to extend the incision beyond the withdrawal limits (3-4 mm) for vascularization of the graft. The gingival epithelium adjacent to the recession was removed by sharp dissection. Immobile connective tissue and periosteum were left at the recipient site. Muscle and loose connective tissue fibers were removed. Measurements were made at the recipient site, and the dimensions of the graft to be harvested were determined. According to the measurements, marking was made on the palatal donor site. The free gingival graft, approximately 1.5 mm thick, was harvested from the palatal donor site at the level of the first and second premolar teeth, using the marked points as a reference. After the obtained free gingival graft was fixed to the recipient site with simple sutures (Neolact, 5-0, PGLA (polyglycolide-co-l-lactide), Setpa, TR) in the coronal, mesial, and distal directions, the adaptation of the graft to the recipient site was increased by placing sling sutures from the periosteum apically (Figure 2). Gentle digital pressure was applied to the graft for two minutes to ensure tissue compatibility and thinning of the clot layer. After the bleeding at the donor site was controlled, it was left for secondary healing. A non-eugenol periodontal dressing was applied to the recipient site (Coe-Pak, GC Dental, USA).

Postoperative Instructions

The patient received routine postsurgical instructions following the surgical procedure, including a 0.12% chlorhexidine mouth rinse (Kloroben, Drogsan, TR) twice a day along with 100 mg flurbiprofen (Majezik, Sanovel, TR) twice a day for five days. The patient was advised to start caring for the wound area the day following the surgical procedure and to remove only the debris on the surface of the teeth (white brushing) with a soft brush for three weeks. In other areas, she was told to continue normal oral care procedures.

Follow-up Procedure

**Figure 3:**
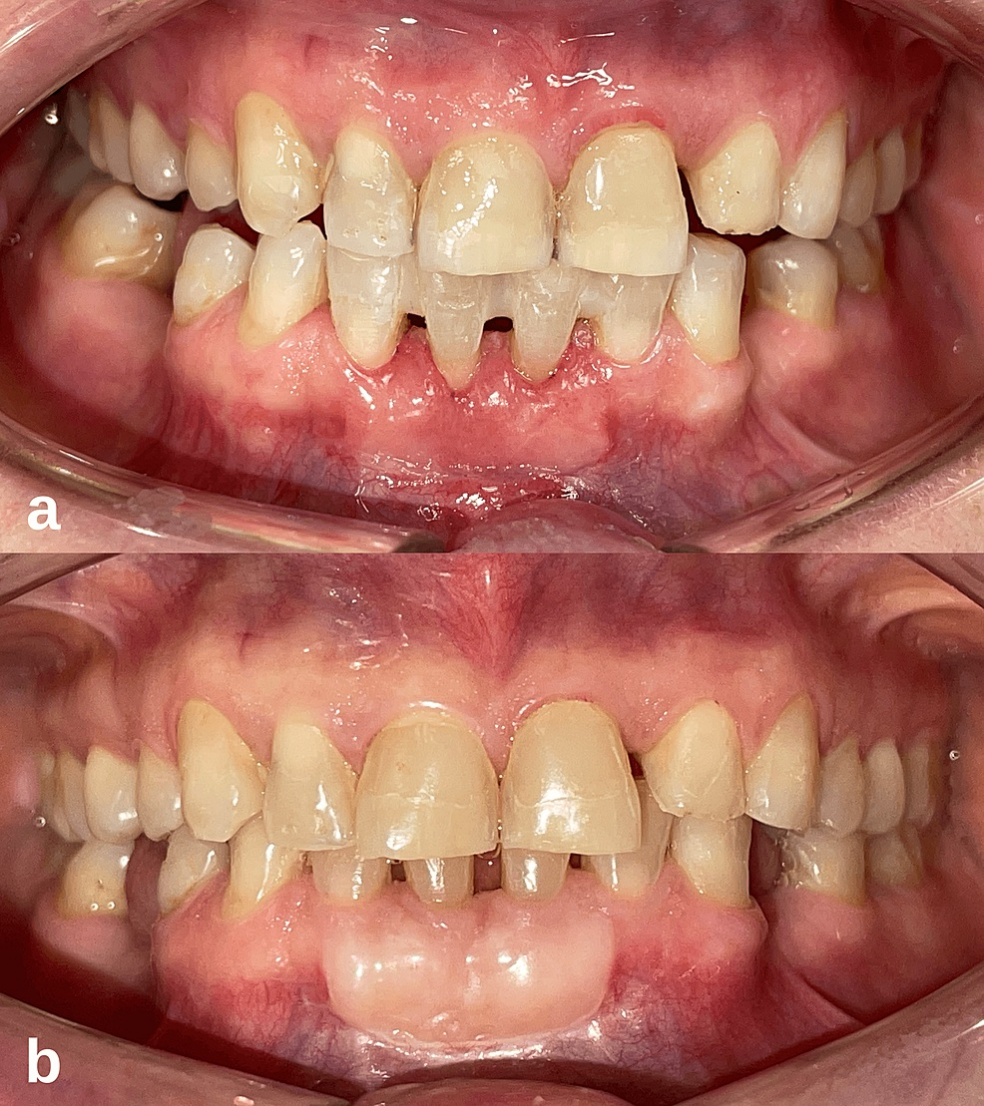
Case 1 postoperative intraoral images of the patient. (a) Intraoral image at the first postoperative month. (b) At the 12-month follow-up, it is observed that there is coverage of the root surfaces with the formation of creeping attachment.

The recovery process of the patient was monitored on days 3, 7, 12, and 30 of the follow-up visits. The periodontal dressing was removed on the seventh day. The sutures were removed on the 12th day (Figure 2). At the four-week follow-up, it was seen that the recovery had progressed smoothly. It was observed that a thick keratinized tissue band formed on the labial side of the mandibular central incisors, but the root exposure continued (Figure 3). During this period, occlusal adjustments were made to eliminate possible trauma to the teeth after surgery and to provide suitable conditions for healing. The patient was advised to continue regular periodontal care appointments. During the 12-month follow-up of the patient, it was observed that creeping attachment formation and complete root coverage were achieved at the relevant site (Figure 3). The clinical attachment level gain of 2 and 4 mm was measured on teeth 31 and 41, respectively. In clinical examination, it was determined that the marginal tissue appeared tight and healthy, and the probing depth was minimal.

Case 2

**Figure 4:**
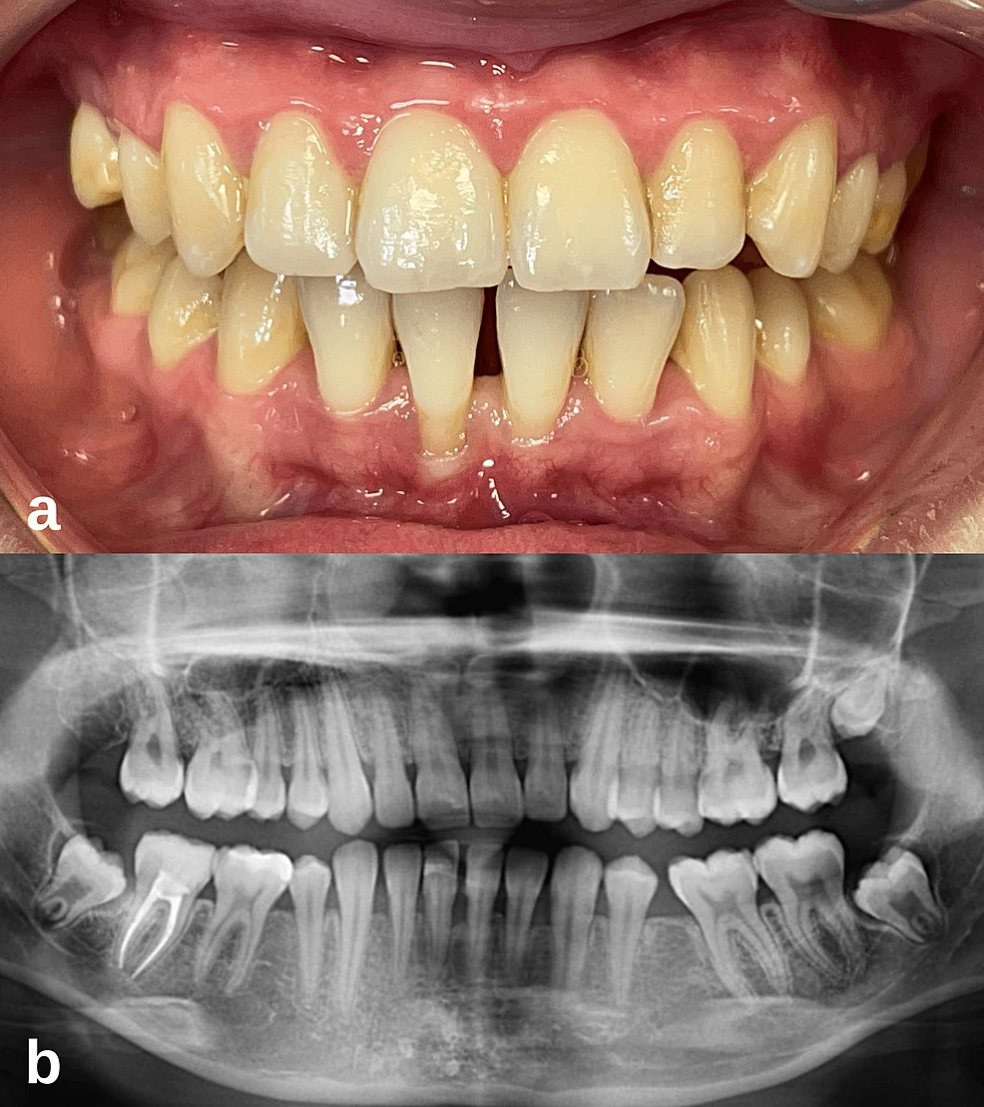
Case 2 initial periodontal examination. (a) Initial clinical intraoral image. (b) Detection of interdental bone resorption in radiographic examination.

A systemically healthy 21-year-old female patient was admitted to our clinic for the treatment of gingival recession in the anterior mandibular region. During the clinical examination of the patient, gingivitis was diagnosed, and it was determined that there was a gingival recession and keratinized gingival deficiency of the mandibular central incisor teeth area (Figure 4). The gingival recession of 2 and 4 mm was measured on teeth numbered 31-41, respectively, and recession defects were determined as Miller’s class III [10]. In addition, 3 and 6 mm attachment losses were measured at the relevant site. The radiographic examination revealed the presence of interdental bone resorption in the mandibular anterior teeth (Figure 4).

**Figure 5:**
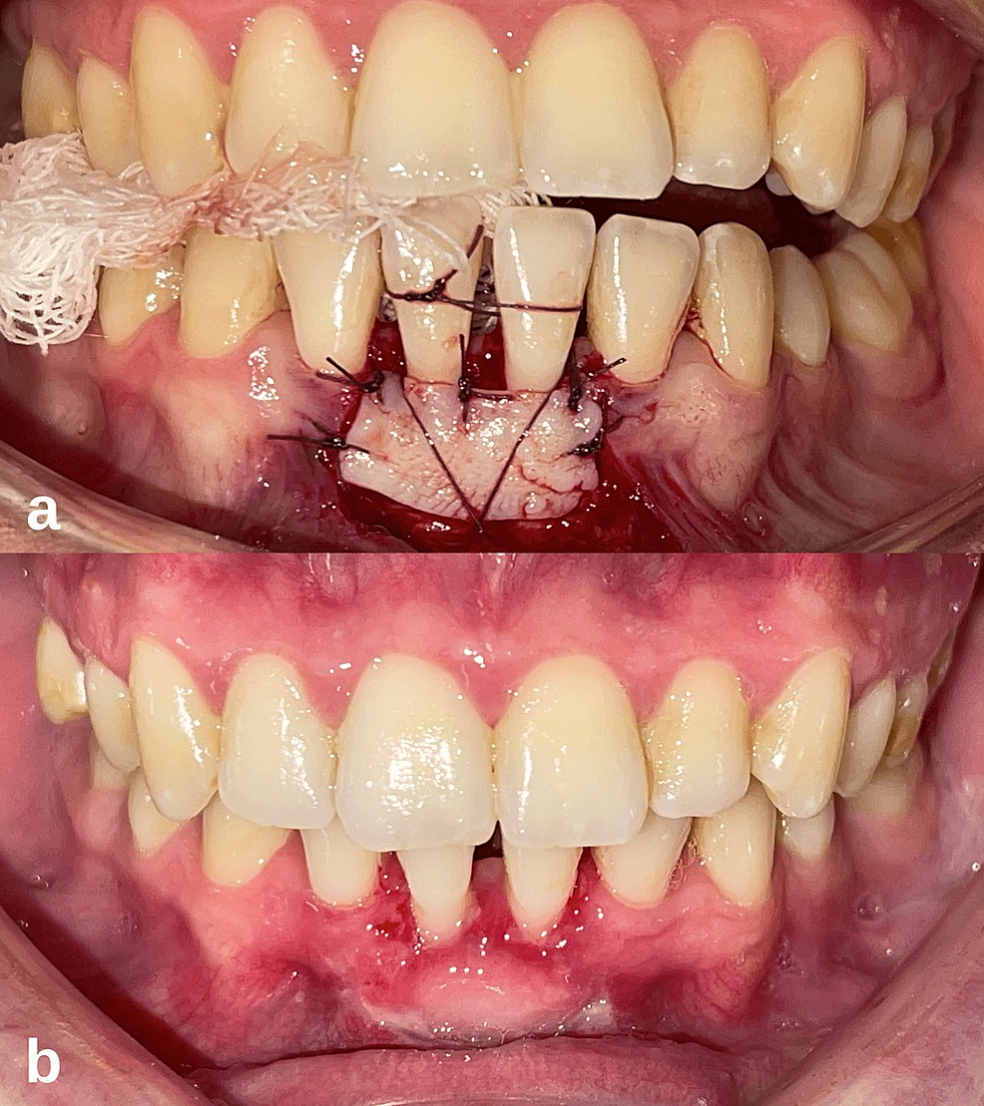
Case 2 intraoral images after the free gingival graft procedure. (a) Fixation of the free gingival graft to the recipient site with simple and sling sutures. (b) Clinical appearance of the surgical site after the sutures were removed on the 12th postoperative day.

The treatment plan, surgical procedures, and postoperative period were performed the same as in the first case (Figure 5). After the initial periodontal treatment of the patient was completed, the free gingival graft was placed on teeth 31 and 41 with a surgical procedure similar to that described in the first case (Figure 5). The postoperative care and follow-up procedures were the same as in the first case.

**Figure 6:**
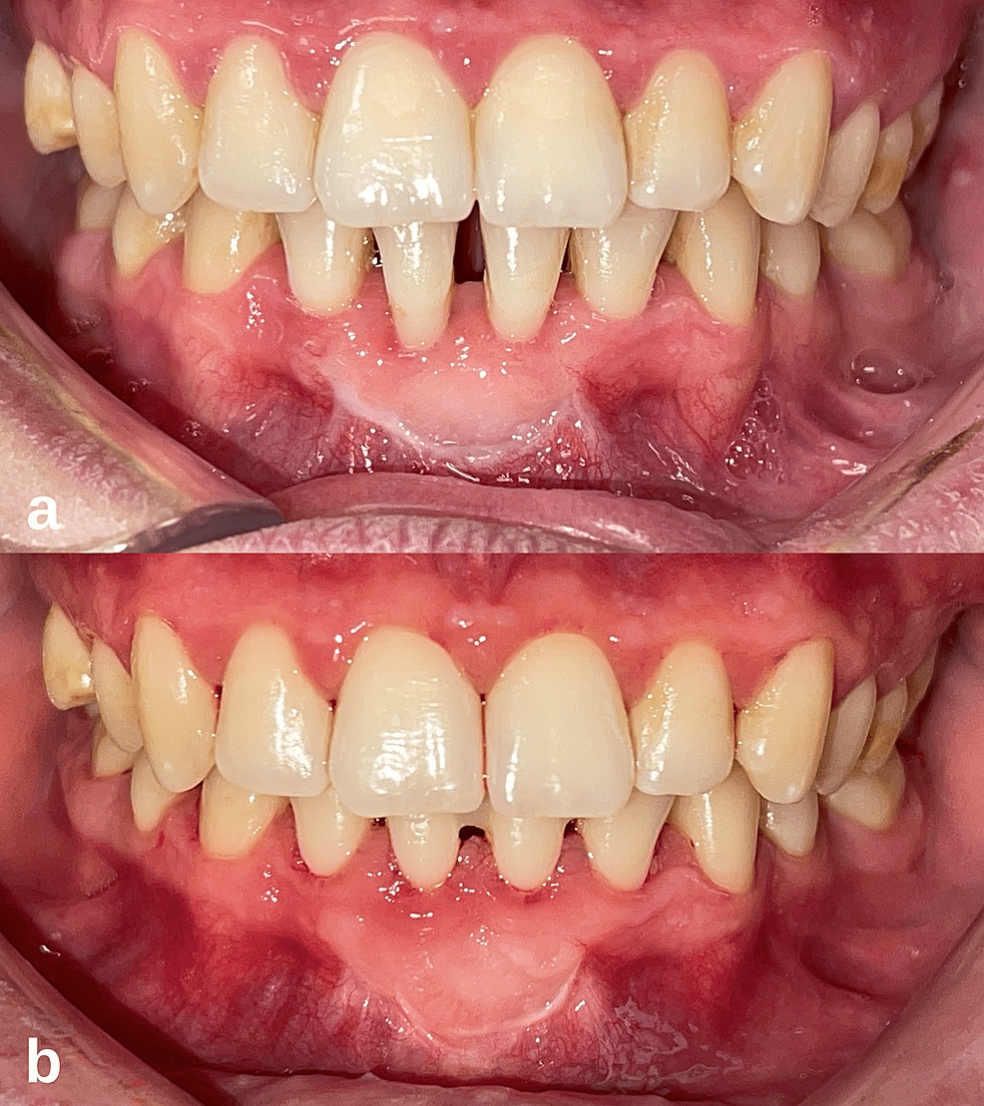
Case 2 postoperative intraoral images of the patient. (a) Clinical intraoral image at the first postoperative month. (b) After the 12-month follow-up, while the width of the keratinized gingiva was preserved, the complete coverage of the root surfaces with the formation of creeping attachment was remarkable.

At the patient’s first month follow-up, it was observed that the healing was uneventful, and adequate keratinized tissue width was formed on the labial side of the mandibular central incisors, but there was no change in the clinical attachment level (Figure 6). The patient was re-evaluated 12 months later. In clinical periodontal evaluation, clinical attachment level gains of 2 and 5 mm were measured on teeth 31 and 41, respectively. It was determined that the gingival tissue had a firm and healthy appearance, and the probing depth was minimal. It was observed that the exposed root surfaces were completely covered with the formation of creeping attachment (Figure 6). It was determined that the width of the keratinized gingiva increased, the gingival recession stopped, and the recession after the surgical procedure was not greater than before.

## Discussion

Treatment of localized gingival recession with free gingival graft is considered to be a preferable method for covering exposed root surfaces [1,9]. Presence or absence of inflammation before the free gingival graft procedure, incorrect measurement of gingival recession, inadequate root surface planing, incorrect preparation of the recipient bed, incorrect preparation of the graft harvested from the donor site, inadequate interdental papilla width, inadequate graft width, inadequate graft thickness, dehydration of the graft, inadequate adaptation of the graft to the root and recipient bed, inadequate graft stabilization, excessive pressure of the sutures on the graft, exposure of the graft to trauma during the healing process, and excessive smoking are the factors that affect the coverage of the root surfaces with the formation of creeping attachment during the free gingival graft procedure [11]. According to our observations, although the lack of coverage on the root surfaces in the early period following the treatment suggests a new surgical intervention, it is recommended to wait for the formation of creeping attachment in cases where the second surgical treatment is not urgent. Monitoring the formation of creeping attachment during the follow-up period may allow the patient to avoid a new surgical procedure and save money and time in terms of postoperative complications. Definitely, in this process, the importance of the application of the oral care procedures correctly and regularly by the patient should be emphasized meticulously. Poor oral hygiene can lead to an increase in the prevalence and severity of periodontal disease by promoting the accumulation of dental plaque [12]. The accumulation of microbial dental plaque may also induce inflammation in the graft placed after a surgical procedure. Particularly, if plaque colonization occurs during the period when creeping attachment formation is taking place, this could negatively impact the movement of the tissue in the coronal direction. In this case report, it was observed that in both cases treated with free gingival graft, the keratinized gingival width increased in the postoperative period, gingival recession stopped, and notably, these positive outcomes were supported by the patients’ good oral hygiene practices. In addition, an average of 3.25 mm clinical attachment level gain was achieved, and complete coverage of the root surfaces occurred.

Creeping attachment was defined by Goldman et al. [8] as the migration of the marginal gingiva in the coronal direction in the postoperative period. The amount of this delayed clinical attachment level gain is thought to be related to the extent of recession, the position of the relevant tooth, the location of the graft, interdental bone resorption, and the patient’s oral care [9]. There are only a few studies in the literature that investigate the formation of creeping attachment and the factors that influence it. In one study, Harris [13] examined the impact of various factors on creeping attachment formation, with subjects aged between 18 and 50. The study found no significant difference in creeping attachment formation between various age groups. Furthermore, smoking was found to have no discernible effect on creeping attachment formation. The study assessed factors such as gingival recession depth, gingival recession width, tooth type, preoperative plaque presence, sensitivity, and high frenulum attachment and found no significant differences between the groups [13]. In both cases presented in this report, the individuals were non-smokers and had no systemic health issues. After the postoperative period, where the ideal graft thickness was achieved, proper wound healing, and diligent oral care practices, the root surfaces were entirely covered with creeping attachment. Thus, the precise impact of smoking and systemic diseases on creeping attachment formation has not been conclusively determined.

It is widely acknowledged that smoking has a detrimental impact on the healing process following periodontal therapeutic procedures [14-16]. Miller [14] reported a 100% correlation between the failure to achieve complete root coverage after soft tissue augmentation and excessive smoking. In a prospective study by Martins et al. [15], involving 15 patients to evaluate the effect of smoking on root surface coverage, it was found that smokers exhibited 82.3% coverage, whereas non-smokers achieved 98.3%. Similarly, in a study conducted by Erley et al. [16], it was determined that smokers (58.84%) had statistically significantly less root coverage than non-smokers (74.73%). In this study, during the postoperative sixth-month control examination, it was observed that smokers had an average depth of 1 mm of gingival recession, and non-smokers still exhibited an average depth of 0.2 mm of gingival recession [16]. Thus, these findings imply that smoking not only directly hinders the success of free grafts but also heightens the probability of unsuccessful cases. This indirect effect is likely to diminish the extent of root surface coverage by influencing the formation of creeping attachment during the postoperative period.

Systemic diseases related to hormones, such as diabetes, may impact the success of mucogingival surgical procedures. Diabetes can interfere with the wound-healing process by affecting blood circulation and may increase the risk of infection by compromising the immune system [17]. These factors can potentially disrupt the vascularization of the graft, negatively influencing the blood supply and adhesion of the graft. This could explain the adverse effects of creeping attachment formation in the subsequent process.

Creeping attachment may occur within 1 to 12 months after the free gingival graft procedure and may continue to progress in the coronal direction in the following period, and its amount cannot be precisely predicted [18,19]. In the study conducted by Bell et al. [7], it was observed that in all cases with localized recessions in the mandibular anterior region, the free gingival graft progressed an average of 0.89 ± 0.46 mm from the gingival margin to the coronal within a 12-month postoperative period. The authors also added that the formation of the creeping attachment does not occur at a constant rate and speed but is completed as a result of successive periods of “recession” and “creep” [7]. Matter and Cimasoni [9] stated in their two-year follow-up study that root surfaces treated with free gingival graft could be covered at a rate of 100%. In both cases presented in this case report, an average of 2.5 mm of creeping attachment formation was measured during the 12-month follow-up period and complete coverage of denuded root surfaces. The limitation of the report is that the patients were not followed up from the first month to the first year. Therefore, it has not been fully interpreted in which period of the 12-month period the creeping attachment occurred.

There are cases of root surface coverage with creeping attachment after free gingival graft surgery in the literature, and these were generally recorded in unrestored mandibular anterior teeth in young individuals [7,9,18]. The presence or absence of interdental alveolar bone is also a factor that plays a role in the healing of free grafts and the formation of creeping attachment [9]. Matter and Cimasoni [9] recorded the best results after free gingival graft procedure in young patients where the recession was on a single root and there was no resorption of the alveolar bone and reported that the possibility of complete coverage of the root surfaces was low in cases where the horizontal bone loss was present. However, although horizontal bone resorption was observed in both cases in this case report, the cases were treated successfully and complete coverage of the root surfaces occurred with the formation of creeping attachment at the end of 12 months. This case report supports that similar and successful results can be obtained in cases with horizontal bone loss as in cases without bone loss.

Although it is still unclear in the literature when and how creeping attachment formation may occur, the common consensus is that it is most likely to occur in narrow recession defects (<3 mm in width) in a single root and depends on the patient’s compliance with oral care procedures [20]. It is a disadvantage that the initial recession widths were not recorded in the patients; however, the fact that creeping attachment formation was observed in two central incisors in both cases can be noted as one of the positive aspects of this case report.

This case report provides important information to evaluate the postoperative results of free gingival graft surgery, but it has some limitations. Because this case report includes data from only two patients, the results may not be suitable for generalization. Longer-term follow-up of the study may help understand the long-term effects of the treatment. This case report focuses only on creeping attachment formation after free gingival grafting. The effect of other variables (e.g., the patient’s general health condition, smoking habit, etc.) on this process has been ignored. In addition, although it was stated that the patients achieved good plaque control, the differences in plaque control methods between patients and the effect of this factor on treatment results have not been determined in detail. In both cases, cases where aesthetic problems were not very obvious and prominent were discussed. Making aesthetic evaluations more objective and measurable may help evaluate treatment results in more detail. In this study, orthopantomographic imaging was utilized for diagnosing interdental bone losses in teeth. While orthopantomographic films were available in our archive, it’s worth noting that the use of periapical images could have provided more specific and useful results for the detailed examination of the region. The examination of patients’ sections with cone beam computed tomography images before the procedure would have allowed for millimetric measurements and a clearer determination of the morphology of bone defects. However, due to the limitation of only having orthopantomographic films for these patients in our archive, this report only includes these images. In future studies, incorporating periapical or cone beam computed tomography images may prove effective in precisely measuring existing defects and improving overall results. Despite all this, the periodontal condition and aesthetics of the teeth treated with creeping attachment formation after the free gingival graft procedure have improved significantly. Increased keratinized gingival thickness and width after the procedure acts as a barrier against plaque retention and thus bacterial retention, allowing the formation of a mucosal environment that will facilitate the patient’s oral hygiene practices.

## Conclusions

This case report emphasizes that free gingival graft is an effective method preferred in the treatment of patients with keratinized gingival deficiency and that creeping attachment formation plays an important role in covering the root surfaces after the surgical procedure. In cases where the aesthetic problem is not very obvious, it is recommended to perform regular control and oral care procedures after free gingival graft surgery and wait before planning a second mucogingival surgery. Especially in patients who maintain good oral hygiene practices, complete coverage of the root surfaces can be achieved through the formation of creeping attachment during the follow-up period. This can help address both aesthetic and functional issues. These results provide dentists with a method they can apply to treat such conditions. However, future studies with a larger number of cases and long-term follow-up may help determine these results more precisely.
